# Hybrid Technique for Cyber-Physical Security in Cloud-Based Smart Industries

**DOI:** 10.3390/s22124630

**Published:** 2022-06-19

**Authors:** Deepak Garg, Shalli Rani, Norbert Herencsar, Sahil Verma, Marcin Wozniak, Muhammad Fazal Ijaz

**Affiliations:** 1 Chitkara University Institute of Engineering and Technology, Chitkara University, Rajpura 140401, Punjab, India; deepak.garg@chitkara.edu.in (D.G.); shalli.rani@chitkara.edu.in (S.R.); 2 Department of Telecommunications, Faculty of Electrical Engineering and Communication, Brno University of Technology, Technicka 12, 616 00 Brno, Czech Republic; herencsn@ieee.org; 3 Department of Computer Science and Engineering, Chandigarh University, Mohali 140055, Punjab, India; sahilverma@ieee.org; 4 Faculty of Applied Mathematics, Silesian University of Technology, 44-100 Gliwice, Poland; 5 Department of Intelligent Mechatronics Engineering, Sejong University, Seoul 05006, Korea

**Keywords:** security, confidentiality, Cyber-Physical System (CPS), smart industrial environment, encryption, decryption, AES, IBE, ABE

## Abstract

New technologies and trends in industries have opened up ways for distributed establishment of Cyber-Physical Systems (CPSs) for smart industries. CPSs are largely based upon Internet of Things (IoT) because of data storage on cloud servers which poses many constraints due to the heterogeneous nature of devices involved in communication. Among other challenges, security is the most daunting challenge that contributes, at least in part, to the impeded momentum of the CPS realization. Designers assume that CPSs are themselves protected as they cannot be accessed from external networks. However, these days, CPSs have combined parts of the cyber world and also the physical layer. Therefore, cyber security problems are large for commercial CPSs because the systems move with one another and conjointly with physical surroundings, i.e., Complex Industrial Applications (CIA). Therefore, in this paper, a novel data security algorithm Dynamic Hybrid Secured Encryption Technique (DHSE) is proposed based on the hybrid encryption scheme of Advanced Encryption Standard (AES), Identity-Based Encryption (IBE) and Attribute-Based Encryption (ABE). The proposed algorithm divides the data into three categories, i.e., less sensitive, mid-sensitive and high sensitive. The data is distributed by forming the named-data packets (NDPs) via labelling the names. One can choose the number of rounds depending on the actual size of a key; it is necessary to perform a minimum of 10 rounds for 128-bit keys in DHSE. The average encryption time taken by AES (Advanced Encryption Standard), IBE (Identity-based encryption) and ABE (Attribute-Based Encryption) is 3.25 ms, 2.18 ms and 2.39 ms, respectively. Whereas the average time taken by the DHSE encryption algorithm is 2.07 ms which is very much less when compared to other algorithms. Similarly, the average decryption times taken by AES, IBE and ABE are 1.77 ms, 1.09 ms and 1.20 ms and the average times taken by the DHSE decryption algorithms are 1.07 ms, which is very much less when compared to other algorithms. The analysis shows that the framework is well designed and provides confidentiality of data with minimum encryption and decryption time. Therefore, the proposed approach is well suited for CPS-IoT.

## 1. Introduction

CPSs are integration of consistent and networked time-sensitive computing frameworks working closely with physical processes, and are deployed in various areas [1,2]. Recently, analyses of CPSs have mainly been focused on security aspects [3,4], authentication and abstraction [5], modelling [6], processing [7] and data management [8]. However, it is crucial to combine cloud computing with CPSs by the design of a methodology, i.e., the Complex Industrial Applications (CIA). More and more security threats have been occurring in recent years due to an increase in the use of technologies that are being used in industrial automation controls, and requirements for interconnection between business networks and industrial networks. The increasing number of smart city applications has helped cloud computing to gain more acceptability in academia and industry. Due to the accession of physical components and the collection of the huge quantity of data from clouds, the network has become a pervasive, suitable, on-demand network for a shared job pool of dynamic computing resources [9,10]. In industrial applications, CPSs use a supervisory control and data acquisition (SCADA) approach to supervise their infrastructure. For example, an application of WebSCADA which is used for smart medical environments improves the patient monitoring and more timely decisions are taken [11]. Security in these types of industries is important as patients’ data is confidential and this requirement is applicable to all real-world applications of IoT. As a result, the security paradigm has become crucial for industrial applications.

A Forbes report (19 December 2018) published reports on five big episodes which give a short depiction of each, including its cause and results: (i) The world’s largest used website Facebook has suffered worst, as its almost 50 billion users were compromised, due to which hackers found a loophole in the website and accessed the ’View As’ privacy tool which enables people to see how their profile looks to the public. All of this happened due to the incredible complexity of their product and failure of imagination; (ii) The second attack occurred on the private information of 500 million customers in the reservation database of Marriott in the Starwood divisions of the hotel company. The hackers obtained names, phone numbers, payment information, mailing addresses, email addresses and passport numbers; (iii) On November 30th, Quora found an enormous breach of data such as IP addresses, user IDs, email addresses, public actions and content like questions, answers, encrypted passwords, blog posts, upvotes and comments of almost 100 million users; (iv) British Airways stated that data of 380,000 customers’ booking transactions had been stolen, such as expiry dates, bank card numbers and CVV codes; (v) One more massive attack was reported on the personal data of 40,000 Ticketmaster customers. The hackers stole the information by attacking a third party. The attack was not on a large scale but it impacted the customers and they also reported that money was stolen.

As per a survey, there are a few problems concerning the ability to attack industrial control systems, such as time-limited authorization, cracking passwords, authentication, fine-grained, collusion attack, malware and DoS attacks. Eventually, security will become comprised among the automation control of various plants, which will damage industrial operations, or even make them fail; it will result in critical health issues, and will compromise safety and the environment. Therefore, the security of smart industrial control devices needs to be improved. Moreover, various studies concerning security in SCADA systems also revealed that the systems are exposed du to their security, which revealed that industries are vulnerable to attacks. This is the case especially where the systems are integrated with IoT and cloud-based systems. Some of the vulnerabilities are described below [12,13,14]:

- Systems can be customized and spoofed/sniffed in communication and data can be modified because the cloud makes the system’s data open.
- Backdoors are open to attackers in cloud servers during industry communication; these are exploited by hackers/attackers.
- SCADA systems which are merged with the cloud servers have the same types of risks as present in traditional architectures.
- The same cloud is shared by many users and their data is categorized at an internal level and hence can be compromised.
- Industrial applications running on the clouds are easily searchable and attackers can sense this type of data. Proper security controls are still not available to secure the data on the clouds.
- Some services are not necessary for the applications and their default factory settings create errors in configurations which lead to problems in IoT systems.
- Most importantly, mismanagement of memory in the validation of input data leads to software errors in IoT-Cloud-based systems. Reliability on third-party services for IoT devices is also a challenge for data tempering and strong encryption.

The above-mentioned reasons for security throw light on the exposure of cloud infrastructure to threats, which has a bad impact on the reliability of the these systems. Threats which take place internally and which are based on CPS-based SCADA systems are the most crucial to take care of [14]. Main threats to the IoT-Cloud are discussed below:

- Advanced Persistent Threats (APTs): These types of threats are made by an unauthorized person who tries to obtain access of the system to gain the data rather than destroy it [15].
- Lack of Data Integrity: Tempering of the data causes this threat and original data are lost; consequently, data integrity is doubtful.
- Man-in-the-Middle (MITM) attacks and spoofing/sniffing attacks are easily carried out in Cloud-IoT systems because they involve impersonation of an original user. Illegitimate access to the network is gained and the intruder tries to monitor the data of that network.
- Outburst Attacks: These attacks are carried out involving the validation message, which keeps on repeating. This affects the performance of the system by incurring a delay concerning important user data.
- Denial of Service (DoS) Attacks: This attack has been very popular for a long time and it still occurs in industries. Because of this attack, the services of industries become delayed and hence users suffer.

### Problem Definition and Motivation

Real-time applications based on IoT-Cloud servers are most prone to security attacks as per the above literature and discussion. Delay is also a major factor for encryption and decryption, where attackers can obtain easy entry to the servers’ channels; delay can give rise to many attacks [15]. Data sensitivity matters a lot, e.g., financial statements can be grouped into bags of words and data can be categorized into three groups. Less delay is expected in the more sensitive data. With the approach proposed in this article, time is taken to carefully address data based on sensitivity; this has not been done in previous literature.

With this paper, we make the following contributions:

(i) In the context of CPSs and cloud-computing environments, security algorithms that are hybrid in nature are proposed.
(ii) In the proposed DHSE with a novel security algorithm, data are communicated for industrial environments to secure data in cloud systems on the basis of sensitivity of data.
(iii) A security analysis of the projected algorithm is conducted by taking the specified amount of data based on the bag of words model.
(iv) The computing time of DHSE is compared with other algorithms and its superiority over the other existing algorithms is validated.
(v) The results are also verified on the basis of the TOPSIS technique to prove the authentication of the algorithms.

## 2. Related Work

A message in which one provides an identity so that the message can only be decrypted with a matching identity is known as Identity-Based Encryption (IBE) and was proposed by Shamir. Later, Sahai and Water proposed KP-ABE. In this method, the encrypted data is matched by attributes and a key is accessed by a monotonic structure which is developed by a combination of various gates, whereas in CP-ABE encrypted data are created by a combination of various gates and the key is accessed by combining the user attributes.

Cloud computing with respect to CPSs has been widely studied in the literature. The authors in [16] presented challenges associated with CPSs and cloud-computing environments. To guarantee consistency [5], architecture was proposed for CPSs using the concept of mapping. In comparison with the SSL authentication protocol, an identity-based protocol for cloud was more trivial and efficient. Afterward, Cheng et al. [17] provided an information security algorithm for cloud computing with the help of IBE. It was known as the benchmarking algorithm for industrial environments, as it helped in maintaining and recording the biometric data of employees, which can be used in the future for various purposes. Shortly thereafter, it was proved that the identity-based data storage protocol [18] supported both inter-domain and intra-domain queries.

**Figure 1:**
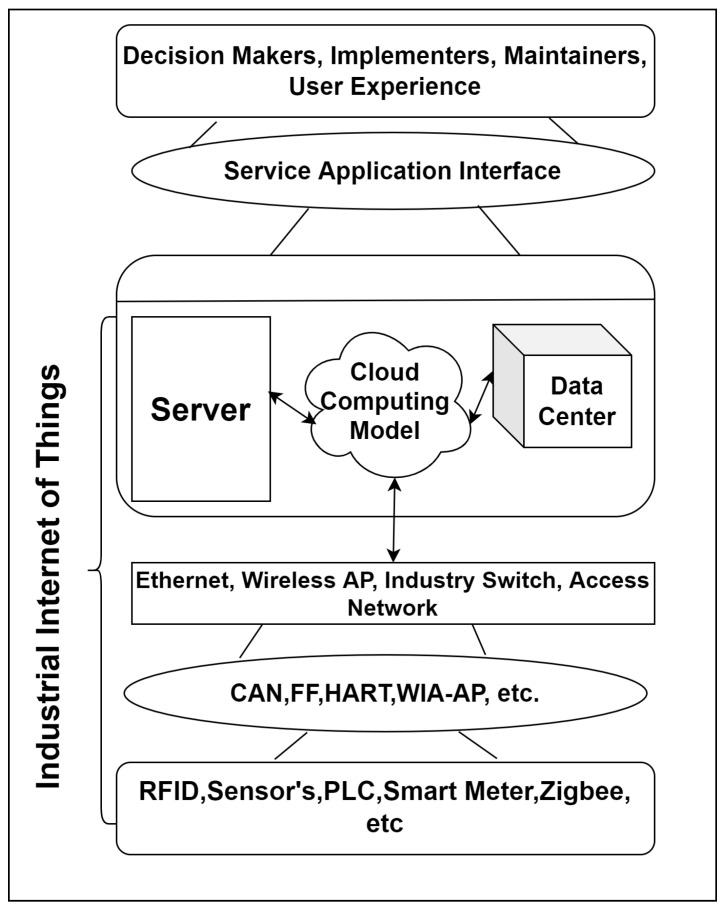
Cloud-based architecture of IOT-CPS.

According to industrial development and deployment, CPSs can be divided into three different domains: communication, control, and computation requirements. In Figure 1, an arrow shows that the connectivity between the three domains results in secure access for industrial CPSs. As studied in the literature, industrial environments involve too many components to collect and store data in a cloud-computing model, e.g., sensors, smartphones, signage, servers, control servers, databases, etc. Once the data has been collected with the support of internet connection, the information is held in the cloud-computing environment. Furthermore, a cloud has layered architecture. Our algorithm DHSE works on the service layer of a cloud-computing environment as it manages databases, servers, message queues, etc.

An access management tree is employed to attain a primary access structure in ABE, which can also help in making a linear access structure, like a threshold structure, OR gate and AND gate. Further, in [19], Rafail Ostrovsky suggested a linear secret sharing mechanism using an access structure with nonlinear properties. Liu [20] constructed a structural access control structure to improve the efficiency in further stages. A new scheme rather than the linear secret sharing scheme named ABE in a finite field was proposed by Balu [21]. It made the scheme much more effective than the traditional schemes. In [22], the authors proposed an ABE scheme that supported multi-values by distributing the earlier situation. In this method, every attribute has two types of status values (1,0), which helps to make the structure more flexible. Article [23] blended a large access control tree from multiple access structures, which reduced encryption costs and ciphertext storage. Article [24] suggested a cipher access control scheme which helps the user attribute revocation mechanisms in a fine-grained manner. Ref. [25] was based on OBDD; it suggested a new structure. It decreased the nodes with comparison of the threshold structure using an access control tree. Ref. [26] increased the problem of over-encrypted data by providing a multi-keyword text quest structure in privacy preservation to ensure the text quest using a similarity-based ranking. In article [27], the writers constructed a scheme to retrieve structures using AND gates on negative and positive parameters. It diminished the encryption/decryption time and the ciphertext size. In this paper, the Dynamic Hybrid Secured Encryption Technique (DHSE) is proposed, which consists of all the three basic techniques, i.e., AES, IBE and ABE, so that DHSE attains minimum encryption and decryption time. As per the above literature, no one has categorized data by sensitivity. The above studies concern security perspectives but lack concepts related to industrial applications. In an IoT-Cloud-based system, it is crucial to consider security on the basis of data. Authors have proposed security algorithms, but verification is carried out on a basis of TOPSIS techniques. This makes our article’s approach better than those of the above-mentioned studies.

## 3. Traditional Methodologies for CPS

In this section, background knowledge related to ABE, AES and IBE is introduced, primarily including special syntaxes, important concepts and basic algorithms. Then, an introduction to our proposed scheme is discussed.

### 3.1. Advanced Encryption Standard (AES)

**Figure 2:**
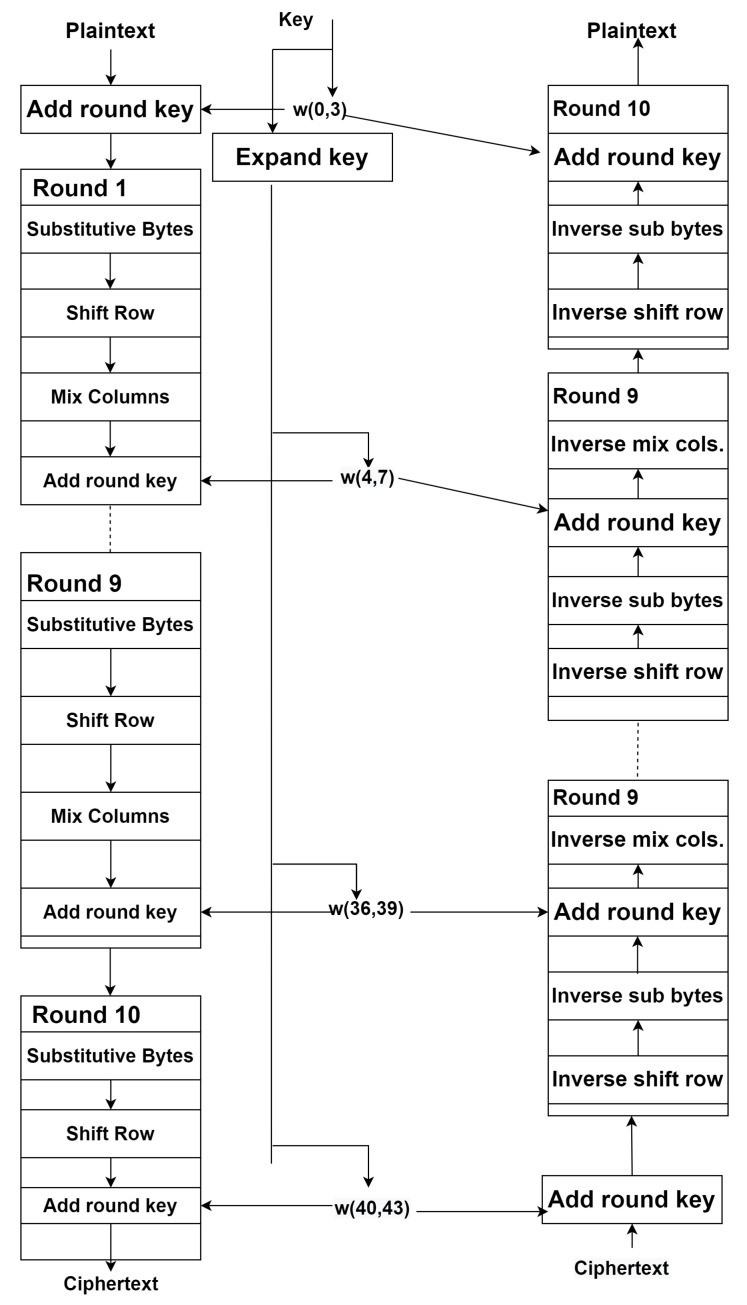
Working Model of Advanced Encryption Standard (AES).

AES uses block cipher format and it can be of 128/156/192 bits. Data blocks are encrypted in 128 bits. Input and output both are in 128 bits. Figure 2 depicts its steps of working.

Pseudocode for AES

Cipher (InBlock [16], OutBlock [16], w [0, ..., 43])

{

BlockToState (InBlock, S)

S ← AddRoundKey (S, w [0, ..., 3])

For (round 1 to 10)

{

S ← SubBytes (S)

S ← ShiftRows (S)

If (round ≠ 10) S ← MixColumns (S)

S ← AddRoundKey (S, w [4 × round, 4 × round + 3])

}

StateToBlock (S, OutBlock);

}

### 3.2. Identity-Based Encryption

**Figure 3:**
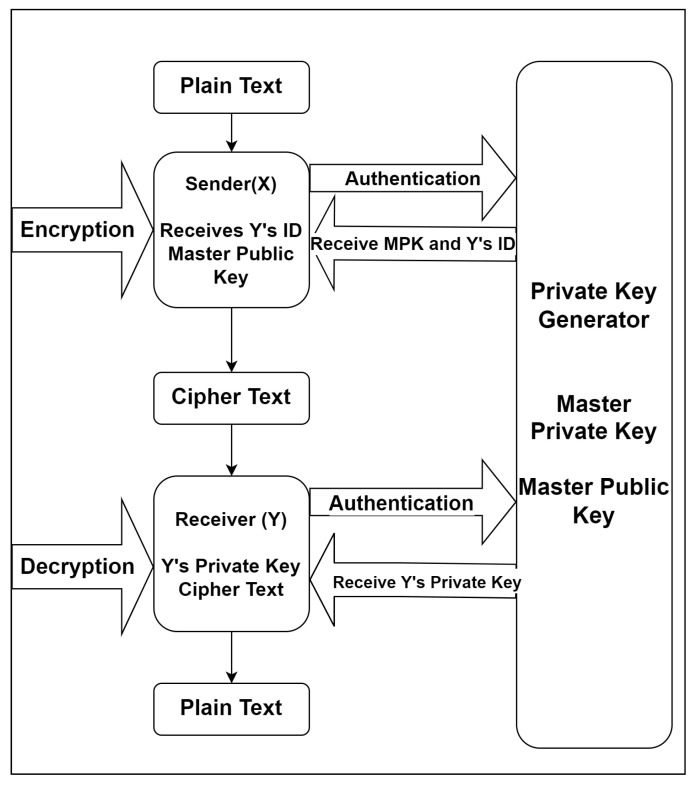
Working Model of Identity-Based Encryption (IBE).

The following randomized algorithms can be used for the encryption scheme based on identity: Setup, Extract, Encrypt, Decrypt as shown in Figure 3.

Setup: This returns arguments and master-keys, which take a security parameter known as k. A characterization of a confined message space M and a characterization of a confined ciphertext space C are among the system parameters. The parameters will be made public, but only the Private Key Generator (PKG) will have access to the master key.

Extract: ε{0, 1}* accepts as input parameters, master-key and an integer ID ε{0, 1}*, having a private key d. ID is a unique string that will be used as a public key, and d is the private decryption key. The Extract algorithm takes a public key and extracts a private key from it.

Encrypt: This considers input parameters, ID and M ϵ M. It returns a ciphertext C ϵ C.

Decrypt: This considers input parameters, ID, C ϵ C and a private key d. It returns M ϵ M.

### 3.3. Attribute-Based Encryption

**Figure 4:**
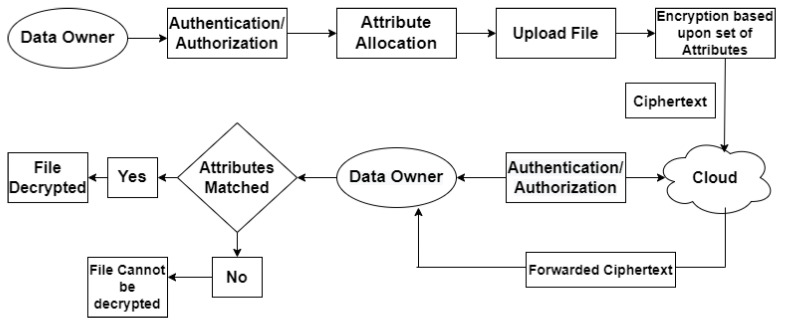
Flow Chart of Attribute-Based Encryption (ABE).

Following are the phases of the ABE scheme (Figure 4):

Setup: This is a stochastic method that only takes the implied security parameter as input. The public parameters PK and a master key MK are the output parameters.

Encryption: This is a stochastic algorithm with a message m, a collection of attributes γ and the public parameters PK as inputs. It generates the ciphertext E as a result.

Key Generation: This is a stochastic algorithm that requires problems A, a master key MK, and public parameters PK as input. It generates D, which is a decryption key.

Decryption: The ciphertext E, which is encrypted using the set γ of attributes, the decryption key D for access control structure A and the public parameters PK are all inputs to this algorithm. If γϵ A, it outputs the message M.

## 4. DHSE: Proposed Approach for Secured Smart Industrial Environment

**Figure 5:**
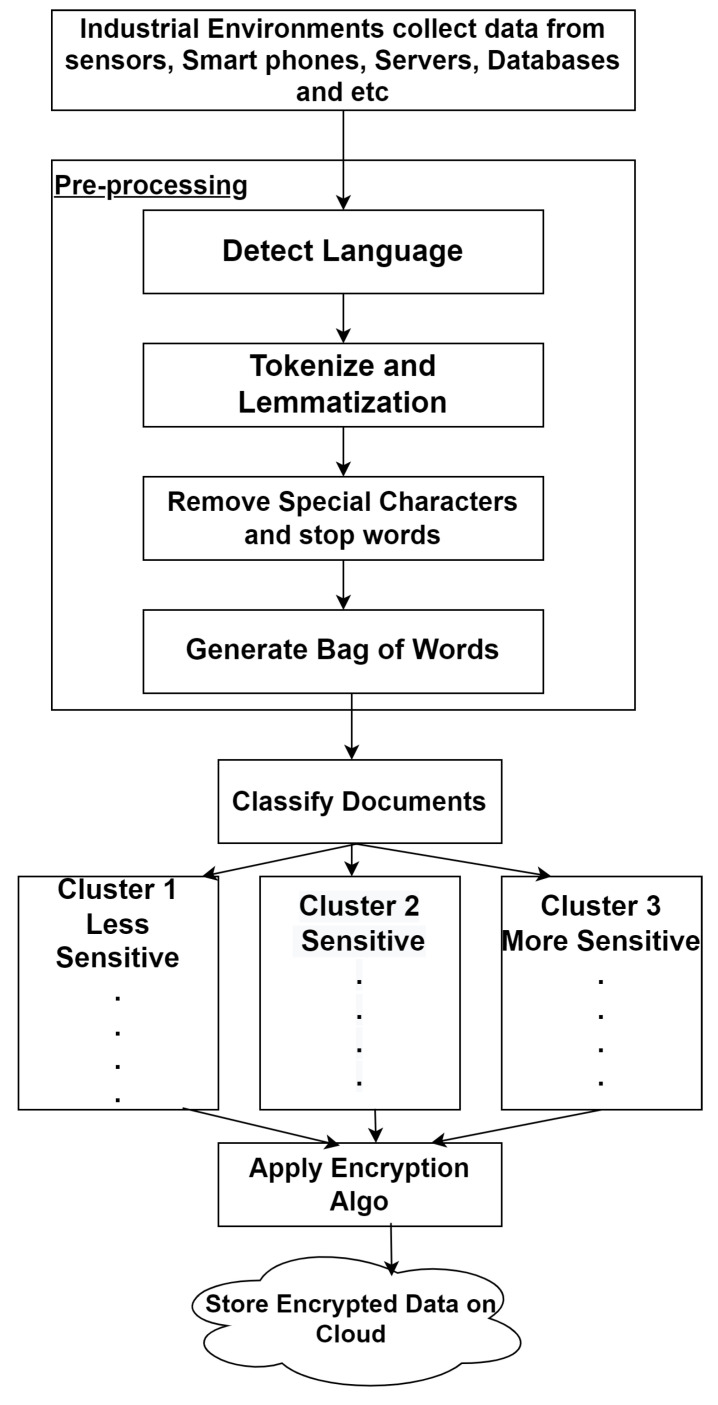
Document Segregation Process.

We built the framework in such a way that it guarantees perfect protection for its providers. The input is split into processing elements before being stored. This method divides the input into three portions, which are then encrypted using various keys and saved in the cloud. Data are separated into three categories in this work, each with a different degree of awareness. The first is the least sensitive, the second is sensitive and the third is the most sensitive (Figure 5). For these various levels of sensitive data, various sorts of keys are used. To address the security parameter, mass remote data augmentation is implemented for data protection. The analysis of the proposed technique with pros and cons is shown in Table 1.

Various properties and principles were discussed in [29]. This method is proposed to provide security to cloud storage at a high level. For this method, secured files were collected and were pre-processed. In pre-processing, various stop words and special characters were removed such that by using various classification techniques, three different bags of words were created. The first contains less sensitive items, the second sensitive and the third more sensitive. Therefore, the input is portioned into three levels of data. After that, a variety of keys are applied to different levels of sensitive data. For the most sensitive data, the strongest key is utilised. Encryption is completed at this stage. Blowfish key is used for less sensitive data, AES is used for sensitive data and ABE is used for more sensitive data for encryption. Finally, data is kept on many cloud servers. The information data were obtained from multiple cloud storage entities and decoded with the keys to decipher them. After that, the data were combined to obtain the first data [17].

The entire process is divided into three phases, A brief description of each phase is as follows:

Phase 1: The input is collected at this phase. As an input, data are entered. Less sensitive, sensitive and more sensitive data are categorised into three groups based on the type of data. Passwords and user IDs fall within the category of more sensitive data. After that, the keys are used to encrypt the data. For less sensitive data, the AES technique is utilised. For sensitive data, IBE is used, and for more sensitive data, ABE is employed. Before the data are delivered to the cloud, the entire process is completed.
Phase 2: Encrypted data are kept in the cloud during this phase. For a proposed system for storing encrypted data, three clouds are used. Data are stored in the cloud using Cloud X, Cloud Y, and Cloud Z.
Phase 3: Encrypted data are obtained from several clouds at this step. The same keys are used to decode them once more. After that, the data are decoded with keys and combined. At the end of the day, we have authentic and protected data.

### 4.1. Data Distribution and Encryption Algorithm

The Data Distribution and Encryption Algorithm [22] is designed to divide data into three categories (Algorithm 1): less confidential, empathetic and more responsive. The data are distributed by grouping named-data packets (NDPs) with named labels. NDPs, pre-stored name lists for more confidential documents (PNL 1) and the pre-stored NMethod 1 show the pseudo-code for the data distribution and encryption algorithm. The following are the steps in this algorithm: Method 1 shows the pseudo-code for the data distribution and encryption algorithm. The pre-stored name lists for confidential documents (PNL 2) are all inputs to this algorithm (PNL2). Each named data packet has a couple of different names and labels. After distribution, the output of this method includes distinct data packets based on their level of sensitivity.

Input two pre-stored name lists (PNL 1, PNL 2), one for more sensitive data packets and the other for sensitive data packets and searchable named-data packets (NDPs).

Step 1: Input two pre-stored name lists (PNL 1, PNL 2), one for confidential datagrams and the other for less vulnerable data packets, as well as searchable designated packets (NDPs).
Step 2: Search each data packet for all NDPs and see if it belongs to PNL 1, PNL 2 or neither of them.
Step 3: If a match is found in PNL 1, the data is encrypted using the IBE algorithm program.
Step 4: If a match is discovered in PNL 2, the data is encrypted using the ABE algorithm.
Step 5: Otherwise, the data packets are encrypted using the AES algorithm.
Step 6: All encrypted information packets, including α, β and γ, should be output and then stored separately in distinct cloud servers.

### 4.2. Data Retrieval Algorithm

As mentioned in Approach 1, this algorithm (Algorithm 2) is designed to retrieve the original data that were first distributed. It accepts the following inputs: α, β, γ, K1, K2, K3. The result of its rule will retrieve original data [20]. The pseudo-code and steps included for data retrieval for this algorithm are described below.

Step 1: We input the encoded data packets produced from Algorithm 1 in this phase, and keys (saved in a dedicated register) are required to access the encrypted items.
Step 2: Then, we create a couple of datasets to store the data when it has been decrypted.
Step 3: Then, using keys and algorithms, we decrypt data from various cloud servers.
Step 4: We combine these decrypted data packets to obtain the original data after we obtain the encrypted data.
Step 5: Output the original data.

Our construction is as follows:

The encryption method to encrypt the less sensitive data AES algorithm is briefly discussed; it is useful for optimization. AES works on the core structure of the 4 × 4 state matrix. It operates in rounds and incorporates a mounted set of transformations that operate on the state matrix. One can choose the number of rounds depending upon the actual size of a key; it is necessary to perform a minimum of 10 rounds for 128-bits keys. For each spherical of the AES formula round, the secret is derived from the first key; this method is named key planning.

To encrypt sensitive data, the encryption method with the public key (ω′) and message (M ∈ G2) is shown in Equation (1). Firstly, the value of s ∈ Zp is chosen dynamically. Afterwards, ciphertext (*E*) can be given as:

(1) $$E=(\omega^{\prime },E^{\prime }=\mathrm{MY}{}^{s},\left\{\mathrm{Ei}=T{}^{s}{}_{i}\right\}I\in \omega^{\prime })$$

Encryption method for more sensitive data (M, γ, PK): To encrypt data (M, γ, PK) under a set of parameters γ, a random value s ∈ Zp is chosen. Based on this, ciphertext is as shown in Equation (2):

(2) $$E=(\gamma ,E^{\prime }=\mathrm{MY}{}^{s},\left\{\mathrm{Ei}=T{}^{\mathrm{si}}\right\}I\in \gamma )$$

Decryption method for sensitive data: The decryption process is shown in Equation (3) and thereafter the values of E′ (encrypted message) and π i ∈$S(e\left(D_{i},E_{i}\right))$δ i,S(0) (decryption) are substituted to obtain the original message (M). ω is the private key, ω′ is the public key and d is the element subset (of set S) of ω∩ω′.

It can be expressed as| ωω′| ≥ d.

(3) $$=\frac{E^{\prime }}{\pi i\epsilon S{\left(e\left(D_{i},E_{i}\right)\right)}^{\Delta }i,S\left(O\right)}$$

(4) $$=\frac{Me{\left(g,g\right)}^{sy}}{\pi i\epsilon_{S}\left(e\left(gq\left(i\right)/ti,gsti\right)\right)\Delta i,S\left(O\right)}$$

(5) $$=\frac{Me{\left(g,g\right)}^{sy}}{\pi_{i}\epsilon_{S}(e\left(g,gsq\left(i\right)\right)\Delta i,S\left(O\right)}$$

(6) =M.

With the help of polynomial exploration in the exponents, the last equality is derived. The polynomial sq (x) has a degree of d−1. It can be explored by the use of d points.

Decryption process for more sensitive data (*E, D*): An algorithm DecryptNode (*E, D, x*) is defined which takes an input ciphertext (*E*= (*γ, E′, {Ei} i*∈γ)) and the private key (*D)*. Th output of an element in group G2 or *⊥* is produced. Assumptions of this algorithm are:

**Assumption** **1.** *The access tree T is enclosed in the private key.*

**Assumption** **2.** *Node x is in T.*

**Algorithm 1:** Data Distribution and Encryption Algorithm
Require: NDP, PNL1, PNL2
Ensure: D, α,β,γ
1. Input NDP, PNL1, PNL2
2. READ: Read data
3. for ∀ NDP do
4. for each data packet do
5. if ∃ a Li ϵ PNL 1 then
6. Key Kh is generated using KeyGenerator
7. IBE Algorithm for encryption using key K1
8. Alpha is generated
9. else if ∃ a Li ϵ PNL 2 then
10. Key Ki is generated using KeyGenerator
11. ABE Algorithm is executed to encrypt the data using key Ki
12. Beta is generated
13. else
14. Key Kj is randomly generated
15. IBE operation for encryption with key Kj
16. Gamma is generated
17. end if
18. end for
19. Values of D is obtained
20. end for
21. Output Alpha, Beta, Gamma


**Algorithm 2:** Data Retrieval Algorithm
Require: Alpha, Beta Gamma, Kh,Ki,Kj
Ensure: D
1. Input Alpha, Beta, Gamma, Kh,Ki,Kj
2. Initialize λ←0, λ′ ←0, λ”←0
3. /* User receives inputs Alpha, Beta, Gamma from different cloud servers*/
4. λ←Alpha decoding with key Kh using ABE algorithm
5. λ′← Beta decoding with key Ki using AES algorithm
6. λ”← Gamma ⨁ Kj
7. D ← Combine λ, λ′ and λ” to obtain original data
8. Output D


### 4.3. Tools and Outcomes

Most of the protection standards do not seem to be enforced, and the certification has not been widely accepted by industrial vendors and users. Several old security systems involve techniques that face distinctive issues, for example, the area unit accustomed test industrial management devices. These issues provide several challenges. The proposed framework was implemented in PyCHARM. The specifications of the computer with which the algorithm is executed are: operating system-Windows 10, CPU-Intel Core i5 @ 2.4 GHz.

#### 4.3.1. Decryption Time

**Figure 6:**
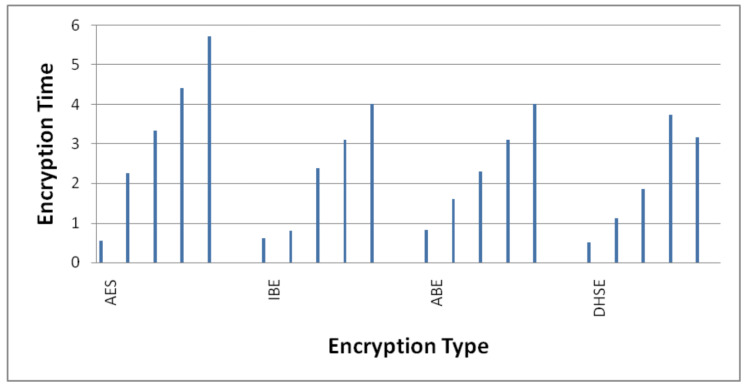
Bitwise encryption time.

**Figure 7:**
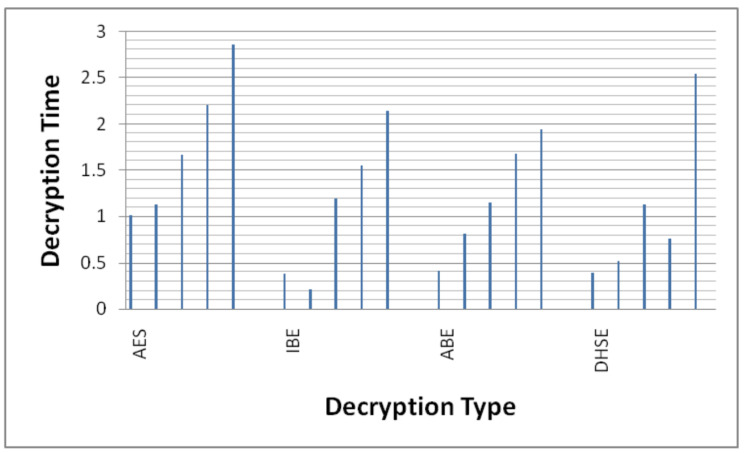
Bitwise decryption time.

The computational time for related works concerning the above process is shown in Figure 6 and Figure 7. The average encryption time taken by AES, IBE and ABE is 3.25 ms, 2.18 ms and 2.39 ms, respectively, whereas the average time taken by the DHSE decryption algorithm is 2.07 ms, which is very less when compared to other algorithms. Similarly, the average decryption times (Figure 7) taken by AES, IBE and ABE are 1.77 ms, 1.09 ms and 1.20 ms and the average times taken by the DHSE decryption algorithm are 1.07 ms, which is very less when compared to other algorithms. DHSE is the only algorithm that takes the minimum time to encrypt data when compared with the other three encryption techniques.

#### 4.3.2. Encryption Time for 20, 40 and 50 KB Data

The percentage decrease in the total time cost of the encryption process for AES, IBE and ABE is 7.08%, 17.82% and 37.56% when the total data size is 10 KB. Moreover, the percentage decrease in the total time cost of the encryption process for AES, IBE and ABE is 51%, 40% and 30.97% when the total data size is 20 KB.

The percentage decrease in the total time cost of the encryption process for AES, IBE and ABE is 44.09%, 21.57% and 19.07% when the total data size is 30 KB. The percentage decrease in the total time cost of the encryption process for AES, IBE and ABE is 15.49%, 20.41% and 11.44% when the total data size is 40 KB.

The percentage decrease in the total time cost of the encryption process for AES, IBE and ABE is 44.86%, 21.42% and 19.10% when the total data size is 50 KB.

#### 4.3.3. Decryption Time for 20, 40 and 50 KB Data

Similarly, the percentage decrease in the total time cost of the decryption process for AES, IBE and ABE is 61.25%, 3.71% and 5.46% when the total data size is 10 KB. The percentage decrease in the total time cost of the decryption process for AES, IBE and ABE is 53.59%, 148.22% and 35.30% when the total data size is 20 KB. The percentage decrease in the total time cost of the decryption process for AES, IBE and ABE is 31.99%, 4.56% and 1.48% when the total data size is 30 KB. The percentage decrease in the total time cost of the decryption process for AES, IBE and ABE is 65.47%, 50.84% and 54.49% when the total data size is 40 KB. The percentage decrease in the total time cost of the decryption process for AES, IBE and ABE is 10.97%, 18.69% and 30.93% when the total data size is 50 KB.

Based on the three different types of encryption/decryption time cost, it is clear that there is a heavy reduction in computation time. The simulation results for encryption in different categories of data are displayed in Table 2.

## 5. Ranking Method for Finding Significant Secured Algorithm

**Figure 8:**
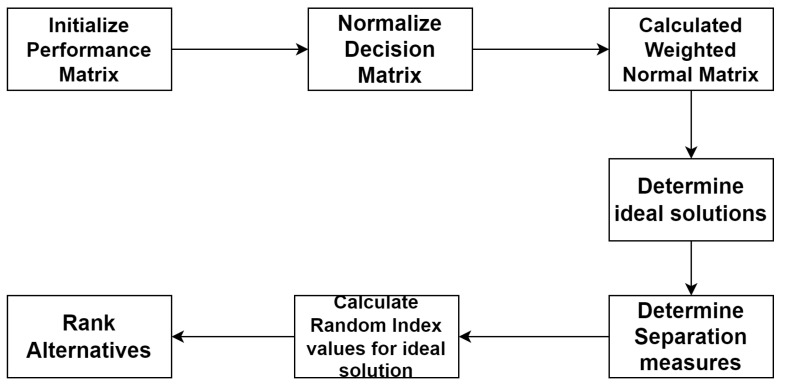
Methodology of TOPSIS.

**Figure 9:**
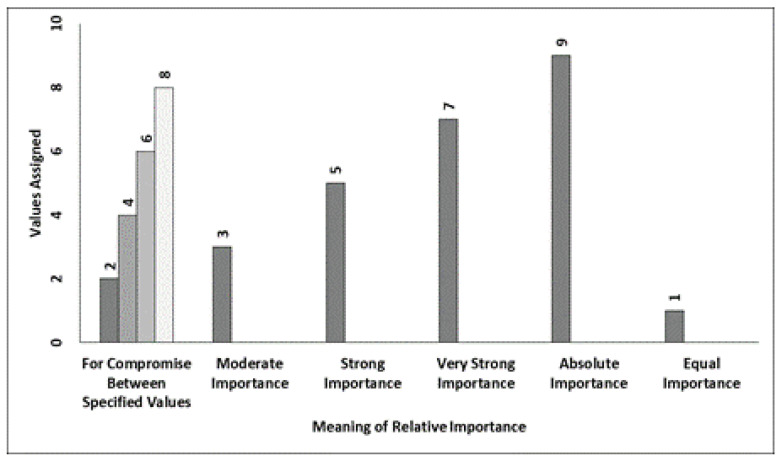
Relative importance values.

**Figure 10:**
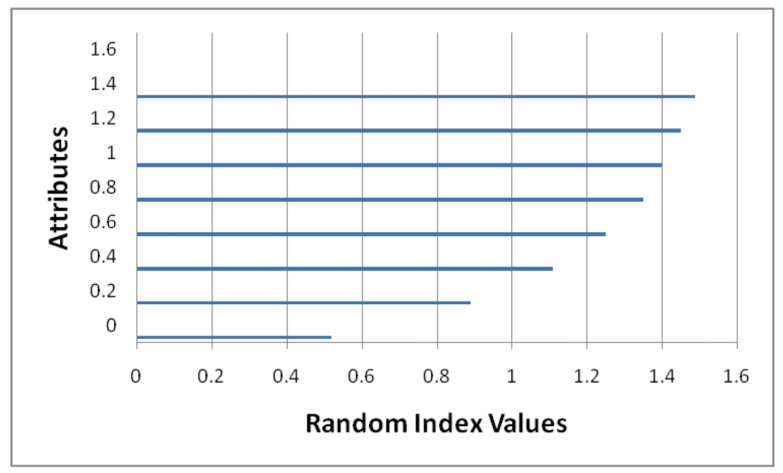
Random index (RI) values.

**Figure 11:**
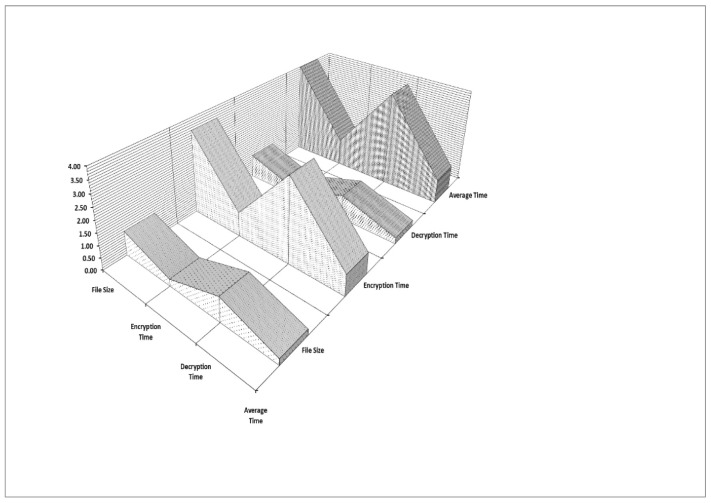
Relative importance of attributes of proposed approach.

**Figure 12:**
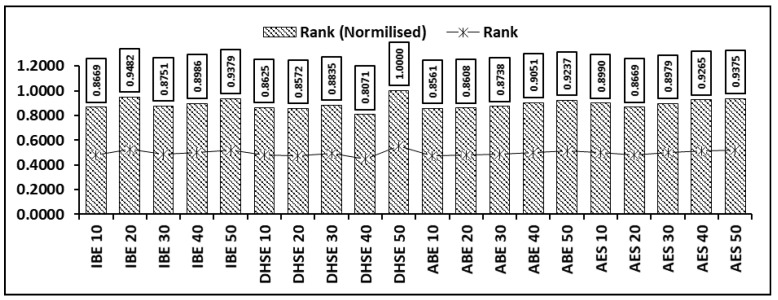
Ranking of encryption techniques.

Decision making is a dynamic process that helps in aggregating significant algorithms and most secure algorithms. In studies, it is presented as Multiple Criteria Decision Making (MCDM) and it is depicted in Figure 8. In the literature concerned with solving very large issues, various techniques such as Simple Additive Weighting (SAW), the Technique for Order of Preference by Similarity to Ideal Solution (TOPSIS) (Figure 8) and the Analytic Hierarchy Process (AHP) [28,30,31,32] are presented by authors; all of these techniques were built on the basis of Zelany’s work [33]. In 2015, Zavadskas [34] proposed a multi-criteria choice technique to build up a profound water seaport in the Klaipeda locale to satisfy financial needs. The proposed approach depends upon AHP and weighted collected total item evaluation technology with fluffy qualities to choose the best strip mall building site, etc. Relative importance values and Random Index (Figure 9 and Figure 10) clearly the importance of CPS in cloud computing. For multi-criteria decision making, authors proposed the Technique for Order of Preference by Similarity to Ideal Solution (TOPSIS); the steps included in the improved TOPSIS. Results of the proposed approach can be observed in Figure 11 and Figure 12. Ref. [24] are given as:

Step 1: The ${\left(OK_{t}\right)}_{n\times o}$ matrix formed by normalizing the matrix ${\left(K_{k}\right)}_{n\times o}$. Values ranging between 0 (most significant) and 1 (least significant) are the parameter values.

(7) $$OK_{t}=\frac{K_{tjk}}{\sqrt{\sum_{j=1}^{n}K_{tjk}^{2}}}$$

Step 2a: To calculate weights, use the method as follows

(8) $$T_{ij}=R_{ij}\times W_{ij}$$

i=1,2,3,⋯,m

j=1,2,3,⋯,n

Step 2b: Construct the matrix using the relative importance scale of AHP [30] (Figure 9) of $T_{Q}$ parameters.

(9) $${K_{t}}_{n\times o}=\;\begin{array}{c} \;\\ {K_{t}}_{1}\\ {K_{t}}_{2}\\ \vdots \\ \vdots \\ {K_{t}}_{n-1}\\ {K_{t}}_{n} \end{array}{\left[\begin{array}{cccc} T_{Q_{1}} & T_{Q_{2}} & \cdots & T_{Q_{o}}\\ T_{Q_{22}} & T_{Q_{22}} & \cdots & T_{Q_{10}}\\ \vdots & \vdots & \vdots & \vdots \\ \vdots & \vdots & \vdots & \vdots \\ T_{Q_{(n-1)1}} & T_{Q_{\left(n-1\right)2}} & \cdots & T_{Q_{\left(n-1\right)o}}\\ T_{Q_{n1}} & T_{Q_{n2}} & \cdots & T_{Q_{\mathrm{no}}} \end{array}\right]}_{n\times o}$$

Step 3: Calculate the geometric mean to obtain the weight of the parameters.

(10) $$HN_{k}={\left[\pi_{k=1}^{0}T_{Qjk}\right]}^{1/0}$$

(11) $$X_{k}=\frac{HN_{k}}{\sum_{k=1}^{o}HN_{k}}$$

Step 4: The normalized $T_{q}$ matrix:

(12) $$OR_{O\times 1}=\frac{RpT_{o\times o}}{X_{o\times 1}}$$

Step 5: The relative normalized $T_{q}$ matrix:

(13) $$SOR_{O\times 1}=\frac{OR_{o\times 1}}{X_{o\times 1}}$$

Step 6: Figure 10 shows the random index (RI) values used for making decisions concerning attributes.

Step 7: Weighted normalized decision matrix:

(14) $$U=\left(u_{n\times o}\right)={\left(x_{k}OK_{tjk}\right)}_{n\times o}$$

Step 8: Separation measures:

(15) $$t_{j+}={\left(\sum_{k=1}^{0}{\left(l_{jk}-l_{jk}\right)}^{2}\right)}^{0.5}$$

(16) $$t_{j_{-}}=(\sum_{k=1}^{0}{\left(l_{jk}-l_{jk}\right)}^{0.5}$$

Step 9: Relative closeness (RC):

(17) $$RC=\frac{t_{j-}}{t_{j_{+}}-t_{j_{-}}}$$

Step 10: Ranking as per $RC_{j}$ = (*i* = 1, 2, ..., *n*). In the Technique for Order of Preference by Similarity to Ideal Solution (TOPSIS), it is stated that the selected solution must have the farthest geometric distance for a negative ideal solution (NIS) and the shortest geometric distance for a positive ideal solution (PIS). The ranking process selects the distance between the shortest and the original distance of the solution. The important attributes, such as the means and the standard deviations, verified the proposed approach.

## 6. Concluding Observations and Future Work

A reliable industrial environment for CPSs requires a secure approach that places a restriction on access to sensitive data. In this article, we proposed a DHSE scheme that can be implemented for any types of industrial data. DHSE is a hybrid scheme which is validated by AES, IBE and ABE algorithms in terms of encryption and decryption time while transferring or sharing the data in CPSs of industrial environments. Moreover, data are classified as per their sensitivity, which was not the case in earlier studies. We compared our scheme to existing algorithms and proved that the proposed approach exhibits 1.5-,1.05- and 1.15-fold reduced encryption time over AES, IBE and ABE, and 1.6-, 1.0- and 1.12-fold reduced decryption time over AES, IBE and ABE. In smart industrial environments, delay in any process is intolerable; therefore, DHSE is suitable for CPSs in smart industrial environments. Future analyses will include applying the approach to medium-sized and small industrial organizations, with the aim of analysing its performance during real-world implementation. Moreover, machine learning and deep learning could be applied for the larger dataset, where over-fitting and under-fitting challenges could be solved for further validation of the proposed work.

## Figures and Tables

**Table 1:** Comparitive Analysis of existing algorithms.

Ref.	Technique Used	Parameters	Pros/Cons	Future Scope
Ahmed et al., 2011 [13]	Vehicular CPS	Traffic load, real-time data, tracking information	Traffic navigation and tracking information attainment	It can offer cloud-computing-based real-time services in order to improve a driver’s safety and degree of comfort.
Cheng et al., 2014 [14]	Hierarchical VCPS and MCC Integration Architecture (VCMIA)	Cost function value, value of risk probability	Detection of vehicles in advance, highly efficient, finds the optimal route	Data priority was not considered in it, which can be a real-time problem for analysis in industry applications.
Rajhans et al., 2016 [15]	Smart cloud-based optimizing workload	Cost, execution time, overall performance delay	The number of input tasks, the amount of available cloud servers	Provides near optimal solutions to task assignments in cloud systems to meet sustainability demands.
Sajid et al., 2016 [28]	Cloud-integrated CPS (CCPS)	I/O number, number of missed jobs, running time	Improve the performance and QoS	Establishment of a prototype for CCPS, information exchange mechanism among the various devices and big data-based system optimization.

**Table 2:** Improvement of DHSE in encryption over AES, IBE and ABE.

Size of Data	Existing Algorithms	Improvement of DHSE
10 KB	AES, IBE and ABE	7.08%, 17.82%, 37.56% respectively
20 KB	AES, IBE and ABE	51%, 40%, 30.97% respectively
30 KB	AES, IBE and ABE	44.09%, 21.57%, 19.07% respectively
40 KB	AES, IBE and ABE	15.49%, 20.41%, 11.44% respectively
50 KB	AES, IBE and ABE	44.86%, 21.42%, 19.10% respectively
